# Transposon Insertion Sequencing Elucidates Novel Gene Involvement in Susceptibility and Resistance to Phages T4 and T7 in *Escherichia coli* O157

**DOI:** 10.1128/mBio.00705-18

**Published:** 2018-07-24

**Authors:** Lauren A. Cowley, Alison S. Low, Derek Pickard, Christine J. Boinett, Timothy J. Dallman, Martin Day, Neil Perry, David L. Gally, Julian Parkhill, Claire Jenkins, Amy K. Cain

**Affiliations:** a Gastrointestinal Bacterial Reference Unit, Public Health England, London United Kingdom; b Center for Communicable Disease Dynamics, Harvard T.H. Chan School of Public Health, Boston, Massachusetts, USA; c Division of Immunity and Infection, the Roslin Institute and Royal (Dick) School of Veterinary Studies, the University of Edinburgh, Midlothian, United Kingdom; d Wellcome Trust Sanger Institute, Hinxton, Cambridge United Kingdom; e The Hospital for Tropical Diseases, Wellcome Trust Major Overseas Programme, Oxford University Clinical Research Unit, Ho Chi Minh City, Vietnam; f Department of Chemistry and Biomolecular Sciences, Macquarie University, Sydney, NSW, Australia; University of Maryland, School of Medicine; Harvard Medical School

**Keywords:** bacteriophage, Gram-negative bacteria, mutagenesis, transposons, whole-genome sequencing

## Abstract

Experiments using bacteriophage (phage) to infect bacterial strains have helped define some basic genetic concepts in microbiology, but our understanding of the complexity of bacterium-phage interactions is still limited. As the global threat of antibiotic resistance continues to increase, phage therapy has reemerged as an attractive alternative or supplement to treating antibiotic-resistant bacterial infections. Further, the long-used method of phage typing to classify bacterial strains is being replaced by molecular genetic techniques. Thus, there is a growing need for a complete understanding of the precise molecular mechanisms underpinning phage-bacterium interactions to optimize phage therapy for the clinic as well as for retrospectively interpreting phage typing data on the molecular level. In this study, a genomics-based fitness assay (TraDIS) was used to identify all host genes involved in phage susceptibility and resistance for a T4 phage infecting Shiga-toxigenic Escherichia coli O157. The TraDIS results identified both established and previously unidentified genes involved in phage infection, and a subset were confirmed by site-directed mutagenesis and phenotypic testing of 14 T4 and 2 T7 phages. For the first time, the entire *sap* operon was implicated in phage susceptibility and, conversely, the stringent starvation protein A gene (*sspA*) was shown to provide phage resistance. Identifying genes involved in phage infection and replication should facilitate the selection of bespoke phage combinations to target specific bacterial pathogens.

## IMPORTANCE

Antibiotic resistance has diminished treatment options for many common bacterial infections. Phage therapy is an alternative option that was once popularly used across Europe to kill bacteria within humans. Phage therapy acts by using highly specific viruses (called phages) that infect and lyse certain bacterial species to treat the infection. Whole-genome sequencing has allowed modernization of the investigations into phage-bacterium interactions. Here, using E. coli O157 and T4 bacteriophage as a model, we have exploited a genome-wide fitness assay to investigate all genes involved in defining phage resistance or susceptibility. This knowledge of the genetic determinants of phage resistance and susceptibility can be used to design bespoke phage combinations targeted to specific bacterial infections for successful infection eradication.

## INTRODUCTION

Antibiotic resistance poses one of the greatest threats to public health, with treatment options to reduce levels of mortality and morbidity from bacterial diseases likely to decrease (1). Thus, alternative treatments that are successful in killing resistant bacteria are desperately needed. Phage therapy, a method where viruses that lyse specific bacteria are administered directly to the patient, has been used successfully in certain countries, including Russian Federation, Georgia, and Poland, for decades (2). There, they have relied on continual *in vitro* testing of isolated phages and clinical isolates. There is now renewed interest globally at the academic and commercial levels, with some recent clinical trials of phage therapy showing promise (3, 4) and a number of startup companies and larger companies formed around this approach. Differential phage killing of bacterial cells has been utilized to classify the relatedness of bacterial strains (called phage typing [5]) for almost 80 years. Despite the clear applications and potential value of phage killing, many mechanisms and factors involved in bacterium-phage interactions at the whole-cell level remain to be discovered. As our knowledge of these processes increases, our capacity to utilize this potential should develop, arguably led by genomic prediction of susceptibility. To this end, methods that more accurately characterize phage susceptibility or resistance pathways for sequenced bacterial isolates are needed in order to advance this key field.

Phage and bacteria have coevolved for at least 2 billion years in a complex arms race between the viral predator and the bacterial prey. In terms of the complexity of phage infection of Gram-negative species, phage are known to interact with outer membrane protein (OMP) receptors as well as with the lipopolysaccharide (LPS) of the outer membrane (6), and hundreds of these specific bacteriophage receptors have been identified across different bacterial species (7, 8). After adsorption to the bacterial cell surface, phage inject their nucleic acid payload, which then leads to complex interactions that result in taking control of the host cells. Lytic phages replicate their genome primarily by subverting the host replication machinery, followed by further manipulation of protein expression to produce new phage, in a process analogous to those seen with human/animal viruses. However, phage infection and replication and lysis of the cell can be prevented through bacterial defense mechanisms. These include clustered regularly interspaced short palindromic repeats (CRISPR), restriction-modification systems, heterogeneity in expression of surface components, and abortive infection (9). The complexity of phage-bacterium interactions and the multitude of mechanisms discovered so far indicate that full-genome screens would likely uncover additional host genes involved in phage susceptibility.

Previous analysis of genome sequencing data highlighted difficulties in elucidating the genetic determinants of phage susceptibility and resistance directly from the genome sequence (10, 11). To overcome these difficulties, we employed transposon-directed insertion site sequencing (TraDIS), a method combining phenotypic fitness selection with genomics to simultaneously assay all genes for their involvement in survival under selective conditions (12, 13). Using TraDIS, we evaluated all nonessential chromosomal genes and identified those involved in susceptibility to Escherichia coli infection with a specific T4 phage. This study built on an initial study establishing that a low-density TraDIS library could be used to confirm the role that the capsule plays in Salmonella enterica serovar Typhi resistance to the Vi phage (14). Other studies have also used transposon mutagenesis techniques to investigate phage phenotypes (15–17) for phages λ, PAP1, and φCbK and largely found associations with LPS or external structures such as receptors/flagellar antigens that were involved in phage infection with less emphasis on internal metabolic/resistance operons.

Taking advantage of a phage typing scheme for Shiga-toxigenic E. coli O157 that has been used at Public Health England (PHE) for over 20 years, we have employed TraDIS in an assay where we exposed an E. coli O157 high-density transposon library to subinhibitory concentrations of a typing phage to which the parent was susceptible. We then sequenced out from the transposon insertions in the input and recovered populations to establish a molecular map of E. coli genes impacting host resistance or susceptibility. The molecular basis for the phage typing scheme has been a mystery since the start of its use. Thus, we aimed to understand the mechanisms defining lytic profiles of standardized strains to better understand the fundamentals of phage typing and perhaps why certain phage types (PTs) are found in particular epidemiological settings. The TraDIS method identified novel genes which were confirmed individually as phenotypically controlling phage infection in the host strain. Identifying genes involved in phage replication will broaden our fundamental understanding of phage infection processes and ultimately facilitate the design of appropriate phage to target bacterial pathogens in phage therapy.

## RESULTS

### Genes involved in phage susceptibility.

**FIG 1:**
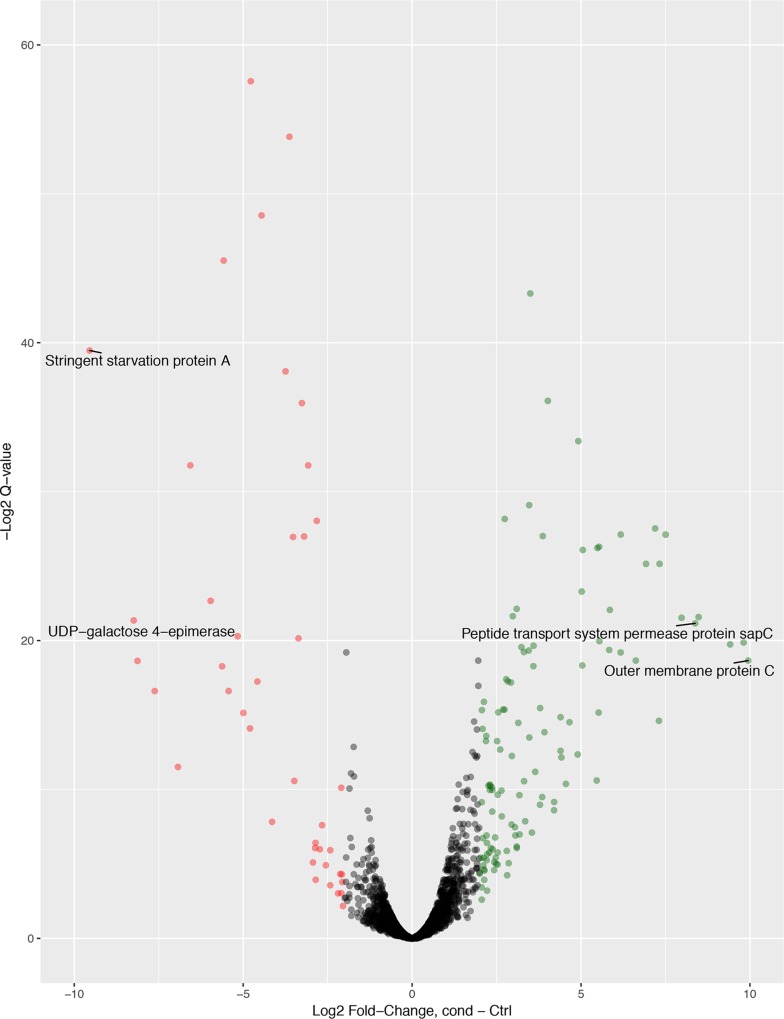
Volcano plot showing changes in prevalence of mutants in the mutant pool compared to the control during the addition of phage selections, as shown by the relationship of log_2_ fold change in selection condition compared to the control (*x* axis), with the *q* value (*y* axis) indicating the false-discovery rate. Colored points show those genes that pass the cutoff criteria of a 1% false-discovery rate and a log2FC of greater than 2 (green) or less than −2 (red). Those genes with the highest cutoff criterion results and discussed in the paper are labeled.

**FIG 2:**
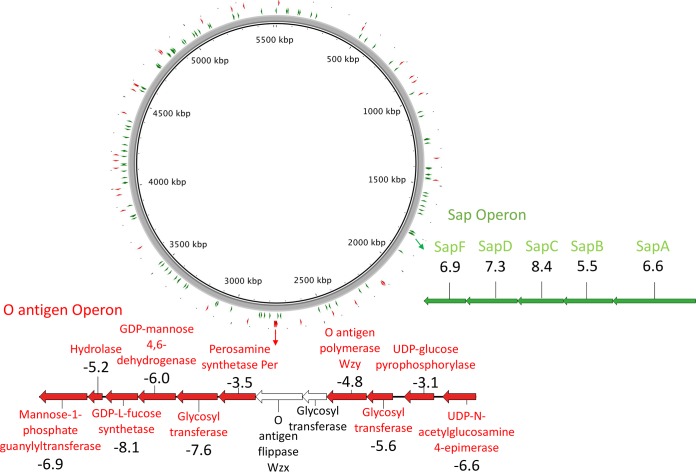
Brig (36) plot showing the location of 114 genes involved in bacteriophage infection (green arrows) and the 44 genes involved in bacteriophage resistance (red arrows) on the chromosome of strain 9000. The location of the sap operon is specified on the chromosome ring, and the names of the genes and log fold change data for each are represented using EasyFig (37). The location of the O antigen operon is specified on the chromosome ring, and the names of the genes and log fold change of each are represented; those genes not implicated in phage resistance are colored white.

Genes that influence phage infection were identified by detecting mutants that were not killed as readily by the selecting phage as was an untreated control (without phage). A total of 114 susceptibility genes, i.e., genes containing insertions that led to the mutants outcompeting the wild-type (WT) control and expanding in the population (>4-fold increase in the number of insertions, or log fold change [LogFC] = >2) (Fig. 1; see also Table S1 in the supplemental material), were identified. The recognized “susceptibility” genes were either located as single genes or clustered in operons (Fig. 2).

10.1128/mBio.00705-18.2 TABLE S1  Genes identified by the TraDIS selections as involved in bacteriophage infection. Here we show the gene locus tag and annotation and the log fold change seen in the TraDIS assay compared to the control with respect to the number of insertions that were observed after bacteriophage selection. Download TABLE S1, DOCX file, 0.1 MB. Copyright © 2018 Cowley et al. 2018 Cowley et al. This content is distributed under the terms of the Creative Commons Attribution 4.0 International license.

Under the conditions used, many of the susceptibility genes with the greatest impact and that had the highest LogFC value had previously been identified as being involved in phage infection. For example, the outer membrane protein (OMP) OmpC and its regulator, OmpR, both showed a dramatic increase in the number of insertions (>9 LogFC) and they both clearly play an important role in susceptibility to this T4 phage. This is to be expected, as OmpC is known to act as a T4 phage receptor without which infection is far less likely to occur (18).

Interestingly, all genes in the *sap* (susceptibility to antimicrobial peptides) operon showed increased numbers of insertions, indicating an expanding mutant population in the presence of the phage (Fig. 2). Thus, the results have shown that each *sap* gene has an effect on the level of phage susceptibility and provide evidence that the entire operon is involved in phage infection. The *sap* operon has previously been associated with resistance to antimicrobial peptides by encoding an ABC transporter to transfer antimicrobials to the cytoplasm to be degraded (19). It has not yet been associated with phage infection. Recent work has shown the *sap* operon to be involved in putrescine export (20). However, SapD has also been shown to be required for activity of Trk system potassium uptake proteins TrkA and TrkH (21). Both *trkA* and *trkH* also displayed increased numbers of insertions and were identified as phage susceptibility genes (Table S1), suggesting that these systems may interact during the period of phage susceptibility. *sapA* has also recently been identified in playing a role in susceptibility to antibody-mediated complement-dependent killing (22) which could involve mechanisms similar to those seen in its involvement with phage susceptibility, such as a general impact on membrane permeability.

One gene (locus identifier [ID] Ecoli9000q_26980), annotated as encoding a hypothetical protein, showed a large and significant increase in the number of insertions compared to the control (LogFC value of 8.48). Its functions were predicted to be involved in polyribonucleotide nucleotidyltransferase activity using extracted gene ontology (GO) terms in the EcoCyc database (23). Polyribonucleotide nucleotidyltransferases are also known as polynucleotide phosphorylases (PNPases) and are widely conserved in all bacteria. PNPases perform core functions in the cell by regulating homologous recombination and mismatch repair (24); this could be essential for the packaging and generation of new phage progeny and the DNA metabolism involved.

### Genes involved in phage resistance.

We showed that 44 genes had a significant reduction in the number of insertions compared to the control (LogFC value of less than −2) (Fig. 1; see also Table S2). These genes are likely to play a functional role in phage resistance, as their inactivation results in cells that are unable to effectively replicate or are killed more efficiently in the presence of T4 phage. Phage resistance modifiers were detected, including stringent starvation protein A and the O antigen ligase, which had a reduced density of insertions, as the *sspA* and *waaL* gene mutants grew at a rate that was lower (by more than 4-fold) than that seen with the control in the presence of phage. Genes with the largest reduction in the number of insertions were mostly involved in outer membrane and replication cellular processes.

10.1128/mBio.00705-18.3 TABLE S2  Table showing the 44 genes highlighted by the TraDIS selections found to be involved in bacteriophage resistance. The table shows the gene locus name, the annotation of that gene, and the log fold change compared to the control with respect to the number of insertions that were observed after bacteriophage selection. Download TABLE S2, DOCX file, 0.1 MB. Copyright © 2018 Cowley et al. 2018 Cowley et al. This content is distributed under the terms of the Creative Commons Attribution 4.0 International license.

Stringent starvation protein A had the largest fold decrease in the number of insertions compared to the control (LogFC of −9.54), indicating that it likely plays an important role in the prevention of phage production through transcriptional regulation. Stringent starvation protein A is a global transcriptional regulator that is associated with RNA polymerase and acts through inhibition of accumulation of H-NS to upregulate multiple stress defense systems (25). Curiously, it has also been shown to be essential for expression of bacteriophage P1 late genes (26) but has never been directly implicated in the infection process.

The 44 resistance genes were found dispersed throughout the chromosome (Fig. 2) but also exhibited clustering in 4 regions, implicating several key operons in providing phage resistance. Significantly, 7 of the top 10 genes implicated in phage resistance were found on the O antigen biosynthesis operon (27) (Fig. 2). This operon contained the O antigen polymerase gene *wzy*, although the O antigen ligase that showed a decrease in the number of insertions (LogFC of −5.93) is located on another region of the chromosome. This may indicate that the O antigen itself blocks phage adsorption in some manner similar to the strategy represented by *Salmonella* capsule-based resistance to Vi phage (14). Interestingly, the O antigen flippase *wzx* gene that is found on the O antigen biosynthesis operon showed only a small decrease in the number of insertions (LogFCof −0.04) which was not below the cutoff that defined confidence in involvement. This means that our cutoff was too stringent to detect its involvement and that the other genes in the operon showed a decrease in the number of insertions which a greater confidence could be assigned to. However, the lipid III flippase protein *wzxE* gene was found on another part of the chromosome and did show a significant decrease in the number of insertions (LogFC of −3.5).

### Confirmation of phenotype conversion with knockout assays.

Genes known to be involved in the synthesis of LPS and extracellular/cell surfaces structures, such as the susceptibility gene encoding OmpC also identified here, have long been associated with phage infection. However, TraDIS has proven to be an extremely sensitive gene identification technique (28, 29) and here, we were able to detect completely novel genes potentially involved in the phage infection cycle (including both genes conferring resistance and genes conferring susceptibility).

To confirm whether these novel genes implicated in phage infection individually affected the phage susceptibility/resistance phenotype for all the T4 and T7 phages of the E. coli O157 typing scheme, a phage plaque-forming assay (see Fig. S1 in the supplemental material) with defined single-gene knockouts was performed on 6 highly significant gene hits. Three potential resistance gene product mutants were selected to confirm increased susceptibility: stringent starvation protein A (*sspA*), O antigen ligase (*waaL*), and GDP-l-fucose synthetase (*fcl*; involved in O antigen biosynthesis [27]). Similarly, 3 mutants were chosen to test the susceptibility phenotype: a hypothetical protein (Ecoli9000q_26980), a putrescine ABC exporter membrane protein (*sapC*), and a transcriptional activator for formate metabolism operons (*fhlA* [30]). In this experiment, we predicted that the resistance gene mutants would have increased susceptibility to the E. coli O157 typing phages compared to the parent strain and that the susceptibility gene mutants would have a greater tolerance than the parent.

10.1128/mBio.00705-18.1 FIG S1  Phage typing lysis plates for the gene knockout mutants of 6 genes identified by TraDIS (Table 2). The plates show the number of viable bacterial cells indicating the change in effective phage lysis compared to the parent strain (1465). A 5-µl volume of a dilution series of E. coli O157 typing phage 13 (starting with undiluted phage) was spotted onto 1.5% agar plates. Plates are labeled with the designation of the gene that had been knocked out followed by the lysis profile of the strain, which is indicated as follows: +, full lysis; (+), partial lysis; −, full resistance. Download FIG S1, DOCX file, 0.8 MB. Copyright © 2018 Cowley et al. 2018 Cowley et al. This content is distributed under the terms of the Creative Commons Attribution 4.0 International license.

Table 1 shows the lytic profiles of the knockout strains compared to the wild type as tested on 14 T4 phages and 2 T7 phages from the E. coli O157 typing scheme (5). The ability to detect a change in lytic profile was hampered by the degree of difference that the knockout would garner in comparison to the WT profile. For example, for some of the typing phages, the WT is already fully lytic, and so any resistance-associated genes are unlikely to produce an observable phenotype change in plate assays as the strain cannot become more susceptible than the fully lytic WT. This occurred for typing phages 3, 7, and 13. Conversely, for one of the typing phages, the WT is already fully resistant, so any susceptibility-associated genes are unlikely to produce an observable phenotype change in plate assays as the strain cannot become more resistant than the WT. This occurred for typing phage 16.

**TABLE 1:**
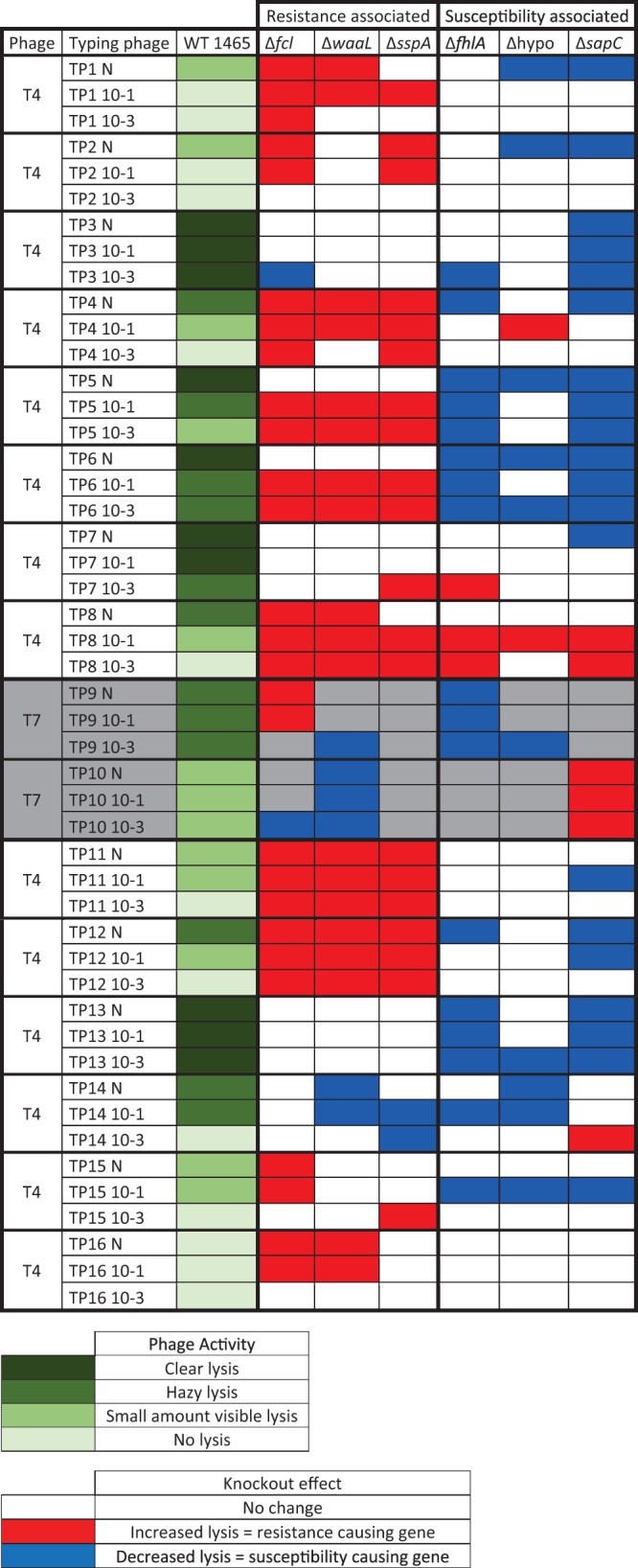
Phage plate plaque assay results for typing phages 1 to 16 of the E. coli O157 phage typing scheme (5)^a^

a Levels of lysis in the wild type (WT) are represented by shades of green, with darker colors representing more-complete levels of lysis, from 5 µl of a dilution series of E. coli O157 typing phage 13 (starting with undiluted phage [N]) spotted onto 1.5% agar plates. The knockout effect is represented by white, red, or blue squares, where white indicates no change, red indicates increased lysis, and blue indicates decreased lysis.

The two mutants associated with O antigens (*fcl* and *waaL*) were more susceptible to all the T4 phages tested (apart from those already fully susceptible plus typing phage 14) and also converted the WT strain to being susceptible to T4 typing phage 16, which was the only phage it was fully resistant to. The stringent starvation protein A knockout also caused the T4 phages to become more susceptible than the WT, apart from those already fully susceptible and typing phage 14. However, the stringent starvation protein A knockout did not convert the knockout strain to become susceptible to typing phage 16. Interestingly, the *waaL* and *sspA* knockouts had an effect on typing phage 14 susceptibility that was opposite the predicted effect and the *fcl* knockout had an effect on typing phage 3 susceptibility that was the opposite the predicted effect.

The *sapC* knockout converted the WT to become more resistant to all the T4 typing phages, apart from typing phage 16, to which was already fully resistant, and typing phages 8 and 14. The hypothetical protein knockout converted the WT to become more resistant to all the T4 typing phages, apart from typing phage 16, to which it was already fully resistant, and typing phages 3, 7, 8, 11, and 12. The *fhlA* knockout converted the WT to become more resistant to all the T4 typing phages, apart from typing phage 16, to which it was already fully resistant, and typing phages 1, 2, 7, 8, and 11. Interestingly, typing phage 8 became more susceptible to all the susceptibility-associated knockouts that are predicted to convert the strain to being more resistant. Similarly, the *sapC* knockout converted the WT to being more susceptible to typing phage 14, the hypothetical protein knockout converted the WT to being more susceptible to typing phage 4, and the *fhlA* knockout converted the WT to being more susceptible to typing phage 7.

The T7 typing phages behaved differently from the T4 typing phages in their lytic profile changes. Typing phage 9 showed increased lysis for the O antigen *fcl* gene but decreased lysis for the other O antigen gene, *waaL*. However, typing phage 9 did display the expected decreased lysis for two of the susceptibility-associated knockout genes, namely, *fhlA* and the gene encoding a hypothetical protein. On the other hand, typing phage 10 displayed effects opposite those expected for resistance-associated genes *fcl* and *waaL* as well as for the susceptibility-associated *sapC* gene. Typing phage 10 is known to specifically bind the O antigen of E. coli O157 (31), so it is intuitive that these knockouts would have the countereffect for typing phage 10. Interestingly, stringent starvation protein A showed no effect on resistance for either of the T7 phage.

## DISCUSSION

TraDIS is a genome-wide mutagenesis methodology that has proven useful in identifying the obvious capsule genes associated with phage susceptibility conversion (14) as well as with other external structures such as receptors/flagellar/antigens (15–17). In this study, we optimized the TraDIS method to successfully investigate bacteriophage-host interactions of lytic T4 and T7 phages and an E. coli O157 strain, thereby uncovering novel genes involved in phage infection and resistance. One advantage of this method is that it also enables identification of genes conferring resistance, and therefore we were able to systematically identify genes that normally hinder phage infection. Several genes involved in E. coli O157 susceptibility and resistance to T4 and T7 phages were identified, and a subset of the genes were confirmed by site-directed deletion, including implication of some in phage interactions for the first time. Thus, these data have contributed to a general understanding of the molecular interactions governing T4 and T7 phage infection of E. coli. Such fundamental biological information may aid the development of phage therapy as well as in identifying potential novel bacterial drug targets in the future.

Clearly, phage infection is a complex process involving and relying on a large number of effector genes that can hinder the process effectively when knocked out. The dispersed locations of these genes and the involvement of multiple functional pathways in bacteriophage infection demonstrate that bacteria have evolved a complex and multipronged defense system to protect the cell against phage infection. This also means that there is a diverse pool of nonobvious genes to investigate for improved understanding of phage susceptibility.

We were able to identify phage susceptibility genes that showed an increase in the number of insertions compared to the control. These were genes that are normally associated with productive phage infection and could be interpreted biologically, as most encoded outer membrane targets/receptors, which represent the primary point of phage contact. The gene whose mutant displayed the highest fitness, based on the log fold change in the number of insertions compared to the control, was *ompC*. This is an established receptor for T4 in E. coli (18). Without this gene, mutants could flourish during phage infection compared to the remainder of the population. OmpC is a universal OMP in E. coli, and bacteriophage that are able to use it as the primary receptor generally have a broad host range across E. coli; this interaction does not always lead to successful lysis, but initial infection and cell adherence are at least possible if the target bacterial cell has *ompC* (32). The mutants that also had an expanded population during phage exposure included transcriptional regulators *ompR* and *envZ*, which are known to control *ompC* (33).

The most commonly detected genes from other studies that used transposon mutagenesis techniques to examine other phages were those corresponding to phage binding sites, such as outer membrane substrate transporter genes in E. coli (15), O antigen genes in Pseudomonas aeruginosa (16), and flagellar genes in Caulobacter crescentus (17). However, using TraDIS, we could drill more deeply into the full mechanisms of phage susceptibility and resistance and, in particular, into what happens after successful adsorption. This study was the first of its kind to successfully identify completely novel phage susceptibility/resistance genes and to confirm them phenotypically using single gene knockouts. Another significant gene of interest (Ecoli9000q_26980) encoded a hypothetical protein which was implicated in polyribonucleotide nucleotidyltransferase activity. The full phenotype conversion was confirmed by the knockout plaque assay in half the tested T4 and T7 phage, confirming an important role in certain examples of phage susceptibility. The importance of *sapC* in phage susceptibility was also successfully confirmed phenotypically in 11 of the 16 tested phages. However, we can speculate that other mutations in the *sap* operon would reflect this phenotype as the TraDIS data implicated the entire *sap* operon as having increased numbers of insertions (Fig. 2). The *sap* operon has previously been associated with antimicrobial resistance, but this is the first time that it has been implicated in phage susceptibility. The exact mechanism underlying the involvement of this operon in both bacteriophage infection and antimicrobial resistance requires further investigation.

Novel phage susceptibility genes investigated here included those associated with infection and efficient lysis specifically involved in transcription and translation (e.g., *fhlA*). Efficient phage reproduction may require taking over the transcriptional and translational apparatus of the host cell for phage products. The exact role that these regulatory genes play in phage infection has yet to be established, but here we established that a large number of genes were involved in complex multistep processes affecting the success of phage infection. These gene roles were not proven phenotypically but demonstrated that, potentially, not all genes identified by TraDIS would necessarily show a measurable altered phenotype in a single-gene-knockout assay. This could be due to functional redundancy that is accounted for by other genes when knocked out.

Many genes showed a decrease in the number of insertions and thus were potentially associated with phage resistance. The gene most significantly affected during bacteriophage infection and which was involved in resistance encoded stringent starvation protein A. Using an *sspA*-directed knockout plaque assay, we were able to confirm that in cells without this gene, the bacteriophage exhibited more-effective infection in 10 of the 16 phages tested (all of which were T4 phages). This result contrasts with its reported effects on another phage (34). This raises the interesting possibility that the genomic landscape of the host may play a role in resistance to some phages and susceptibility to others. Thus, comprehensive high-throughput studies that are broader in scope are needed to assess a number of phages and bacterial strains using the TraDIS method that we developed here to gain a complete atlas of phage infection processes.

Many of the resistance genes identified, specifically, the somatic (O) antigen synthesis genes, are involved in LPS synthesis. This may be expected, as the removal of O antigens would enable bacteriophage to gain easier access to the OMPs and receptors. The external surface of a bacterial cell is the first point of contact for an infecting bacteriophage via the outer membrane or LPS. The LPS may be an initial adsorption target, but LPS O chains might also occlude OMPs. If a cell is able to occlude receptors by the presence of LPS O chains or some other mechanism, then this could increase their resistance to phage. Conversely, an insertion that inhibits O antigen production may result in the mutant becoming more susceptible, as the phage can access irreversible adsorption sites on OMPs.

To further investigate the function of the proteins encoded by the 6 phenotypically confirmed genes implicated in phage susceptibility/resistance, we predicted protein function *in silico*, using homology comparisons to characterized Pfam domains (shown in Table 2). The homology comparisons were performed using an E value of 1.0 and the multiple-sequence alignments and hidden Markov models (HMMs) of the Pfam database (35).

**TABLE 2:** Table showing the Pfam database (35) and GO term assignments by EcoCyc (23) for protein function predictions for 6 of the most significant implicated TraDIS genes^a^

Gene locus ID	Gene	Gene product annotation^b^	Protein functional domains (Pfam accession no.)	GO term function (GO accession no.)^c^	Predicted role during phage infection (TraDIS LogFC)^d^	Potential role(s) in phage resistance/susceptibility
Ecoli9000q_26980		Hypothetical protein	3′ Exoribonuclease family, domain 1 (PF01138); 3′ exoribonuclease family, domain 2 (PF03725); polyribonucleotide nucleotidyltransferase, RNA binding domain (PF03726); K homology domain (PF00013); S1 RNA binding domain (PF00575)	Biological processes, including response to heat (GO:0009408), RNA processing (GO:0006396), mRNA catabolic process (GO:0006402), and RNA phosphodiester bond hydrolysis, exonucleolytic (GO:0090503); molecular function, including 3'–5′-exoribonuclease activity (GO:0000175), polyribonucleotide nucleotidyltransferase activity (GO:0004654), protein binding (GO:0005515), cyclic-di-GMP binding (GO:0035438), identical protein binding (GO:0042802), nucleic acid binding (GO:0003676), RNA binding (GO:0003723), transferase activity (GO:0016740), nucleotidyltransferase activity(GO:0016779), and metal ion binding (GO:0046872); cellular components, including cytosol (GO:0005829), membrane (GO:0016020), and cytoplasm (GO:0005737)	Increases susceptibility (8.48)	Packaging and generation of new phage progeny and DNA metabolism
Ecoli9000q_3240	*sapC*	*sapC* Peptide transport system permease protein SapC	N-terminal TM domain of oligopeptide transport permease C (PF12911); Binding-protein-dependent transport system inner membrane component (PF00528)	Biological processes, including putrescine transport (GO:0015847) and transport (GO:0006810); molecular function, including putrescine transmembrane transporter activity (GO:0015489); cellular components, including plasma membrane (GO:0005886), integral component of plasma membrane (GO:0005887), membrane (GO:0016020), integral component of membrane (GO:0016021), and ATP-binding cassette (ABC) transporter complex (GO:0043190)	Increases susceptibility (8.38)	Inner membrane transport of phage material into cell
Ecoli9000q_19710	*fhlA*	*fhlA* Hydrogenase-4 transcriptional activator	GAF domain (PF01590); Sigma-54 interaction domain (PF00158); bacterial regulatory protein, Fis family (PF02954)	Biological processes, including DNA-templated transcription and initiation (GO:0006352), regulation of DNA-templated transcription and initiation (GO:2000142), phosphorelay signal transduction system (GO:0000160), transcription, DNA-templated (GO:0006351), and regulation of transcription, DNA-templated (GO:0006355); molecular function, including DNA binding (GO:0003677), nucleotide binding (GO:0000166), ATP binding (GO:0005524), transcription factor binding (GO:0008134), and sequence-specific DNA binding (GO:0043565); cellular components, including intracellular (GO:0005622) and cytosol (GO:0005829)	Increases susceptibility (7.31)	Binding Sigma-54 to interrupt the phage shock protein pathway to prevent phage defense
Ecoli9000q_27560	*sspA*	*sspA* Stringent starvation protein A	Glutathione *S*-transferase, N-terminal domain (PF02798); glutathione *S*-transferase, C-terminal domain (PF00043)	Biological processes, including response to stress (GO:0006950), response to starvation (GO:0042594), and positive regulation of transcription, DNA (GO:0045893); molecular function, including bacterium-type RNA polymerase core enzyme binding (GO:0001000) and protein binding (GO:0005515); cellular components, including cytosol (GO:0005829)	Increases resistance (−9.54)	Protein-protein interaction mediation in resistance mechanisms
Ecoli9000q_13690	*fcl*	*fcl* GDP-l-fucose synthetase	NAD-dependent epimerase/dehydratase family (PF01370)	Biological processes, including metabolic process (GO:0008152), nucleotide-sugar biosynthetic process (GO:0009226), colanic acid biosynthetic process (GO:0009242), "*de novo*" GDP-l-fucose biosynthetic process (GO:0042351), and oxidation-reduction process (GO:0055114); molecular function, including protein homodimerization activity (GO:0042803), GDP-l-fucose synthase activity (GO:0050577), catalytic activity (GO:0003824), oxidoreductase activity (GO:0016491), isomerase activity (GO:0016853), and coenzyme binding (GO:0050662); cellular components, including cytoplasm (GO:0005737) and cytosol (GO:0005829)	Increases resistance (−8.13)	Occlusion of outer membrane protein phage receptors by O antigens
Ecoli9000q_31840	*waaL*	*waaL* O antigen ligase	O antigen ligase (PF04932)	Biological processes, including lipopolysaccharide core region biosynthetic process (GO:0009244) and lipopolysaccharide biosynthetic process (GO:0009103); molecular function, including O antigen ligase activity (GO:0008754) and ligase activity (GO:0016874); cellular components, including plasma membrane (GO:0005886), membrane (GO:0016020), and integral component of membrane (GO:0016021)	Increases resistance (−5.93)	Occlusion of outer membrane protein phage receptors by O antigens

a Data indicate the European nucleotide archive (ENA) accession number, the number of sequenced reads generated per sample, the percentage of those reads that mapped to reference strain 9000, the total number of unique insertion sites (UIS), the number of transposon mutants, and the rate of insertion (average number of base pairs between insertions).

b Data were determined with Prokka.

c Data are from EcoCyc.

d Fold change compared to untreated control (see Table S1).

The gene encoding the hypothetical protein (Ecoli9000q_26980) was previously predicted, using gene ontology from the EcoCyc database (23), as having polyribonucleotide nucleotidyltransferase activity. More specifically, searching the Pfam database (35) revealed that the gene encoded a protein related to 3′ exoribonucleases, polyribonucleotide nucleotidyltransferase (PNPase), showing K homology (present in proteins that bind RNA) and S1 RNA binding capacity. This suggests a potential role in the nucleotide synthetic activity that phages require for replication of their DNA to package into new phage progeny. Importantly, PNPases have been shown to be involved in virulence of E. coli O157 by regulating the expression of Shiga toxin through lambdoid prophage activation and involvement with phage replication (36). The proven involvement with lambdoid phage replication and the implications of this TraDIS experiment provide evidence that PNPases are important for T4, T7, and lambdoid phage replication. Further links of PNPase to virulence reported in E. coli O157 (36) might provide clarity as to why certain phage types are more commonly associated with severe infections. The roles of both *fcl* and *waaL* in phage resistance were already strongly predicted to be expressed via the occlusion of OMPs by O antigens (37) using GO term analysis, through their roles in O antigen biosynthesis, specifically as a GDP-l-fucose synthetase and an O antigen ligase, respectively, using our *in silico* method.

A change in phage resistance was not observed phenotypically for the *fhlA* knockout, which could have been due to regulatory redundancy. However, we were able to predict a potential functional role for this gene, as protein Pfam domain hits were to the GAF domain, Sigma-54 interaction, bacterial regulatory protein, and the Fis family. E. coli phage shock protein F is known to bind Sigma-54 (38). Thus, this transcriptional activator may be interrupting the phage shock protein pathway that normally protects the cell and may thereby be acting as a phage susceptibility gene.

The Pfam predictions for the *sapC* gene were the N-terminal TM domain of oligopeptide transport permease C and the binding-protein-dependent transport, inner membrane component. These results provide further evidence that the Sap operon is involved in putrescine transport and is likely to be the inner membrane transporter of phage material into the cell and important in phage susceptibility. The *sspA* gene had protein function prediction hits of a glutathione *S*-transferase (GST). However, it is known that despite the characteristic fold of GST, SSpA does not have GST activity and cannot bind glutathione (39). SSpA is known to have a surface-exposed pocket, which suggests that it mediates protein-protein interactions (39), but its mechanism for phage resistance remains undetermined, and a more thorough investigation of its function is needed.

As the associated genes recognized to be involved in susceptibility or resistance were found independently in all four replicates (1-µl phage, 200-µl phage, and 3-h and 5-h time points), a high confidence can be assigned to them. Now that the TraDIS method has been successfully validated to identify the drivers of bacteriophage survival in susceptible strains, the next step would be to use the same method for typing phages to which the library strain exhibits resistance.

In this study, we have used the TraDIS method to successfully identify several novel genes involved in maintaining the balance of phage/bacterial survival. This analysis has shown that bacteriophage susceptibility or resistance involves fluctuating molecular states that genomic techniques such as TraDIS are able to capture. As the situation regarding antibiotic resistance becomes increasingly important, we need to investigate novel methods of killing pathogenic bacteria, and phage therapy holds promise for killing certain dangerous bacterial species, such as E. coli O157. During this study, a novel gene involved in phage resistance was identified, the stringent starvation protein A gene, and the entire *sap* operon was implicated in phage infection for the first time, providing evidence for the molecular basis for the E. coli O157 phage typing scheme. Further work identifying unknown host factors involved in successful phage lysis, or in resistance to it, would be helpful in the development of phage therapy or novel drug targeting.

## MATERIALS AND METHODS

### Producing competent cells of library strain 1465.

An E. coli O157 phage type 32 (PT32) Stx2a/Stx2c knockout of strain 9000 (10) termed strain 1465 was used to produce the library. Two single colonies of strain 1465 were inoculated into separate 20-ml volumes of lysogeny broths (produced in-house at PHE) each and left to propagate overnight at 37°C. Each 20-ml overnight culture was added to 300 ml of LB broth and incubated at 37°C until a mid-log phase (optical density [OD] = 0.5) was achieved. The cultures were then recentrifuged at 3,000 relative centrifugal force (rcf) for 10 min at 4°C in 6× 50-ml aliquots. The supernatant was decanted, and the cells were suspended in 25 ml of ice-cold 10% glycerol. The cells were centrifuged again at 3,000 rcf for 10 min at 4°C. Subsequently, the supernatant was decanted and the cells were suspended in 12.5 ml ice-cold 10% glycerol and centrifuged at 3,000 rcf for 10 min at 4°C. The supernatant was discarded, and the cells were suspended in 6.25 ml ice-cold 10% glycerol. The supernatant was removed, and the cells were suspended in 0.5 ml of ice-cold 10% glycerol and centrifuged at 9,000 rpm for 10 min. Finally, the supernatant was removed and the cells were suspended in 50 µl of ice-cold 10% glycerol and kept on ice before electroporation.

### Electroporation of transposome into competent cells.

A 0.5-µl volume of EZ-Tn*5* (KAN-2) Tnp transposome was added to each of the 50-µl competent cell aliquots on ice. The mixture of transposome and competent cells was transferred into 0.1-cm-path-length electroporation cuvettes and electroporated (with settings of 1.4 kV, 25 µF, and 200 Ω) in a Gene Pulser Xcell electroporation system (Bio-Rad). Cells were immediately recovered in 1 ml of Super Optimal Broth with catabolite repression (Life Technologies) after electroporation. This was repeated for each of the 6 aliquots of competent cells. The recovered cells were then incubated for 2 h at 37°C in a shaking incubator.

We have confidence that our Tn*5* transposon inserts in an unbiased manner; the manufacturer’s instructions of the Epicentre Tn*5* transposon kit used (http://www.epibio.com/docs/default-source/protocols/ez-tn5-transposase.pdf?sfvrsn=4) state that the Tn*5* should insert randomly, and although Tn*5* transposons are traditionally thought to have a slight AT preference, they have been shown to unbiasedly saturate E. coli chromosomes overall (40). We are confident that all genes of greater than ~600 bp and containing 1 insertion every 47 bp would be assayed with our method. There is an extremely low (<0.1%) random chance of a gene of an ~600-bp gene not containing any transposons, if nonessential (as first estimated previously [12]), and because we used duplicate cultures, this probability decreases to almost zero. Thus, we believe we have achieved insertions in, and thus can assay, all nonessential genes.

### Recovery of transformed cells.

Transposon-containing colonies were recovered from each of the 6 electroporated cultures on lysogeny broth plates containing kanamycin (4 mg/liter) and incubated overnight at 37°C. Colonies of transformed cells were counted and harvested and then stored at −80°C in a final concentration of 20% glycerol after the optical density (OD) had been recorded for that batch. This method was repeated 30 times to produce 30 different batches, and >1,000,000 mutant colonies were harvested. Batches were pooled in an equimolar manner according to the OD at 600 nm (OD_600_) in the final library.

### TraDIS sequencing and library density.

TraDIS-specific sequencing was performed on a HiSeq 2500 machine using 50-bp single-end reads to produce transposon-directed reads as described previously (41). To determine that insertion sites of each transposon that had randomly inserted into the genome of each mutant, a TraDIS library was prepared using specifically designed TraDIS adapters for the Tn*5* transposon (see Table S3 in the supplemental material). This method increased the enrichment of genuine transposon-chromosome junctions by preventing hybridization of the reverse primer until the transposon-specific forward primer had generated a complementary strand (41). This ensured that the first 10 bp of every read consisted of transposon sequence and that the remaining sequence was downstream of where the transposon was inserted. These reads were then mapped using SMALT (WTSI), and insertion quantification was performed by the use of a PacBio-sequenced reference for strain 9000 (RefSeq accession number NZ_CP018252) to determine the location of the transposon insertions. The reference strain was annotated with Prokka (42) to identify the genes in the insertions were found. Genes essential for survival, which are unable to tolerate any transposon insertions as defined by having less than 10 reads mapped per gene without selection, are not measurable with TraDIS and were thus not assayed in this study. Analysis of the sequencing data showed that the library had >110,000 unique transposon insertion sites throughout the genome with a density of one insertion for every 47 bp of the genome, providing multiple unique insertions in every viable gene and functionally significant noncoding regions. The multiple sites in each locus together act as independent indicators (similarly to “biological replicates”) (43).

10.1128/mBio.00705-18.4 TABLE S3  Table showing the primer sequences used to amplify the transposon sites in the mutant library for TraDIS-specific sequencing. Download TABLE S3, DOCX file, 0.05 MB. Copyright © 2018 Cowley et al. 2018 Cowley et al. This content is distributed under the terms of the Creative Commons Attribution 4.0 International license.

### Bacteriophage selections on library.

The library was interrogated with T4 bacteriophage 13 of the typing phages (5), which was fully lytic when exposed to E. coli O157 PT32 strain 1465. A pool of the library was cultured overnight, with 10^6^ cells added to 10 ml of LB broth. Following incubation for 18 h, 100 µl of the library was inoculated into 10 ml of LB broth and allowed to propagate to mid-log phase. A 100-µl volume of the log phase culture was then inoculated into three 10-ml aliquots of LB broth. Then, 10 µl and 200 µl (multiplicity of infection, ~10) of bacteriophage were inoculated into two of the broths and into one used as a control that was prepared without bacteriophage selection but cultured in parallel under the same conditions. DNA extractions were taken at both the 3-h and 5-h time points using a Wizard genomic extraction kit (Promega). DNA extractions for the controls and the selections were used for TraDIS-specific sequencing (see Table 3) on a HiSeq 2500 platform, generating 14.6 million reads (reads per sample detailed in Table 3). The sequences from all samples were submitted to the European nucleotide archive (ENA).

**TABLE 3:** Results of TraDIS-specific sequencing performed on bacteriophage selections and controls^a^

Sample	Accession no.	Total no. of reads	% mapped	Total no. of UIS	Total sequence length/ total no. of UIS
3_control	ERS939248	4,381,188	80.51252309	3,154,390	89.42522903
3_P13_10µl	ERS939253	4,749,002	81.18015111	3,346,328	86.79931532
3_P13_200µl	ERS939256	4,556,057	80.67339807	3,335,990	90.76224736
5_control	ERS939257	5,011,685	80.23327484	3,647,652	90.71416616
5_P13_10µl	ERS939262	5,194,424	80.37141365	3,858,691	92.42745576
5_P13_200µl	ERS939265	4,140,930	81.17835848	3,219,525	95.77532791

a Designations represent gene locus, gene name, annotation as provided by Prokka (42), protein function prediction hits, LogFC versus the control, and potential role in phage resistance/susceptibility. UIS, unique insertion sites.

### Bioinformatics analysis.

Transposon-directed sequencing reads had transposon tags removed and were then mapped to the genome sequence (sequenced using PacBio technology) of strain 9000 (10) (see Table 3). A change in the number of reads that mapped to each gene between the control and the selections was measured using LogFC (log_2_ fold change), calculated from log counts per million using in-house TraDIS analysis scripts (https://github.com/sanger-pathogens/Bio-Tradis) (41). This approach automatically determines appropriate read mapping parameters from the length of the first read in the fastq file. LogFC was used as a measure of comparison of fold changes in read numbers compared to the LB control; ≥2 and ≤−2 were used as cutoff values for genes with different numbers of insertions (41). False-positivity (*P*) and false-discovery (*q*) values were given for each calculation as a measure of statistical significance in terms of the *P* and *q* rates (44). Only those that had a *q* value of <0.01 and a *P* value of <0.05 were included (see Fig. 1). The data determined for those genes that were implicated independently in all four replicates (10 µl phage, 200 µl phage, and 3-h and 5-h time points) were averaged for the two concentrations to give a consensus list of the genes involved in phage susceptibility and resistance that were present at both time points and both concentrations.

### Gene deletion.

Deletion of individual genes was achieved by replacing the target genes with a chloramphenicol (Cm) acetyl transferase (*cat*) cassette using the standard Lambda Red-mediated gene replacement method (45). Briefly, the *cat* cassette was amplified directly from template plasmid pKD3 using the gene knockout primers described in Table S4. The PCR products (~1 kb) were subjected to agarose gel purification and transformed by electroporation into E. coli strains carrying helper plasmid pKD46. The chloramphenicol-resistant (Cm^r^) transformants were selected on LB agar plates containing 25 µg ml^−1^ of Cm. The deletion mutants were verified by colony PCR using an upstream primer of the target genes with C1, a primer that anneals in the *cat* cassette (Table S4). These PCR products were then sequenced to confirm the insertion of the *cat* cassette in the correct location.

10.1128/mBio.00705-18.5 TABLE S4  Table showing the primers used to verify the deletion mutants by colony PCR using an upstream primer of the target genes with C1, a primer that anneals in the *catA* cassette. Download TABLE S4, DOCX file, 0.1 MB. Copyright © 2018 Cowley et al. 2018 Cowley et al. This content is distributed under the terms of the Creative Commons Attribution 4.0 International license.

### Phage plate plaque assays.

The parent strain (ZAP1465) and each of the deletion mutants were cultured in liquid LB until an OD_600_ of ~1.0 was reached. A 250-µl volume of culture was added to 3 ml of molten top agar and poured on top of an LB agar plate. Once set, 5 µl of a dilution series (starting with undiluted phage) of E. coli O157 typing phages 1 to 16 was spotted onto the plates in the manner shown in Fig. S1 in the supplemental material. The plates were incubated overnight at 37°C and then photographed to show lysis profiles, and the results are summarized in Table 1.

### Accession number(s).

The sequences from all samples were submitted to the European nucleotide archive (ENA), and accession numbers are given in Table 3.
