# Supplementary material for: Transposon Insertion Sequencing Elucidates Novel Gene Involvement in Susceptibility and Resistance to Phages T4 and T7 in Escherichia coli O157
Source: mBio. 2018 Jul 24;9(4):e00705-18. doi: 10.1128/mBio.00705-18 (PMC6058288; doi:10.1128/mBio.00705-18)
Supplement: TABLE S1 [file mbo004183993st1.docx]

| Gene locus | Annotation | LogFC vs the control |
| --- | --- | --- |
| Ecoli9000q_16260 | Outer membrane protein C | 9.948519808 |
| Ecoli9000q_29070 | Osmolarity sensor protein EnvZ | 9.812073088 |
| Ecoli9000q_29080 | Transcriptional regulatory protein OmpR | 9.413818163 |
| Ecoli9000q_26980 | hypothetical protein | 8.479335941 |
| Ecoli9000q_3240 | Peptide transport system permease protein sapC | 8.379641001 |
| Ecoli9000q_28120 | Trk system potassium uptake protein TrkA | 7.975556825 |
| Ecoli9000q_34640 | Potassium uptake protein, TrkH | 7.504031269 |
| Ecoli9000q_3230 | Peptide transport system ATP-binding protein SapD | 7.324206894 |
| Ecoli9000q_19710 | Hydrogenase-4 transcriptional activator | 7.305550369 |
| Ecoli9000q_35390 | 6-phosphofructokinase | 7.192950724 |
| Ecoli9000q_3220 | Peptide transport system ATP-binding protein SapF | 6.923231391 |
| Ecoli9000q_3260 | Peptide transport periplasmic protein sapA | 6.620230847 |
| Ecoli9000q_36030 | Elongation factor Tu | 6.174319841 |
| Ecoli9000q_10280 | Lipid A biosynthesis (KDO)2-(lauroyl)-lipid IVA acyltransferase | 6.172719132 |
| Ecoli9000q_27370 | Nitrogen regulatory protein | 5.853102337 |
| Ecoli9000q_34500 | Ubiquinone/menaquinone biosynthesis methyltransferase ubiE | 5.832565746 |
| Ecoli9000q_54460 | Ribonuclease E | 5.542857839 |
| Ecoli9000q_20540 | Pyridoxine 5'-phosphate synthase | 5.534946763 |
| Ecoli9000q_3250 | Peptide transport system permease protein sapB | 5.520564259 |
| Ecoli9000q_21070 | SsrA-binding protein | 5.484968276 |
| Ecoli9000q_14240 | Galactitol permease IIC component | 5.464163309 |
| Ecoli9000q_47320 | Lipoyl synthase | 5.051970875 |
| Ecoli9000q_50850 | Cytidylate kinase | 5.037706786 |
| Ecoli9000q_t350 | tRNA | 5.019387592 |
| Ecoli9000q_24250 | Protein visC | 4.919457267 |
| Ecoli9000q_28660 | Catabolite gene activator | 4.899718743 |
| Ecoli9000q_8120 | Ribonuclease T | 4.658085247 |
| Ecoli9000q_3150 | protein yciW | 4.552476946 |
| Ecoli9000q_34580 | 3-octaprenyl-4-hydroxybenzoate carboxy-lyase | 4.418526722 |
| Ecoli9000q_17060 | Phosphate acetyltransferase | 4.394529392 |
| Ecoli9000q_24260 | 2-octaprenyl-6-methoxyphenol hydroxylase | 4.392847702 |
| Ecoli9000q_28080 | Protein smf | 4.203530306 |
| Ecoli9000q_54090 | protein YmdC | 4.201746198 |
| Ecoli9000q_27430 | Aerobic respiration control sensor protein ArcB | 4.016808495 |
| Ecoli9000q_36020 | Pantothenate kinase | 3.916358073 |
| Ecoli9000q_34510 | protein yigP | 3.868346125 |
| Ecoli9000q_13490 | Exodeoxyribonuclease I | 3.849851565 |
| Ecoli9000q_24220 | Glycine cleavage system H protein | 3.788714647 |
| Ecoli9000q_16960 | reductase | 3.785724884 |
| Ecoli9000q_42320 | Chaperone protein skp | 3.643697255 |
| Ecoli9000q_41690 | Aconitate hydratase 2 | 3.59398253 |
| Ecoli9000q_19900 | Inosine-5'-monophosphate dehydrogenase | 3.58964449 |
| Ecoli9000q_1170 | Protease 7 | 3.546143167 |
| Ecoli9000q_24070 | Lysyl-tRNA synthetase | 3.497873184 |
| Ecoli9000q_31710 | SecB protein | 3.468424177 |
| Ecoli9000q_54110 | Glucans biosynthesis protein G | 3.465076834 |
| Ecoli9000q_54720 | HTH-type transcriptional regulator ycfQ | 3.451890337 |
| Ecoli9000q_29660 | High-affinity branched-chain amino acid transport ATP-binding protein LivG | 3.34686467 |
| Ecoli9000q_36230 | protein yjaG | 3.314032794 |
| Ecoli9000q_41120 | RNA polymerase-associated protein rapA | 3.309043081 |
| Ecoli9000q_41070 | Chaperone surA | 3.236827539 |
| Ecoli9000q_1790 | transport protein | 3.179788013 |
| Ecoli9000q_10730 | Ferritin-like protein 2 | 3.175587189 |
| Ecoli9000q_41650 | Pyruvate dehydrogenase E1 component | 3.143582779 |
| Ecoli9000q_40500 | Aerobic respiration control protein ArcA | 3.098701665 |
| Ecoli9000q_45750 | Hemolysin expression-modulating protein Hha | 3.096482664 |
| Ecoli9000q_14440 | outer membrane usher protein yehB | 3.096437589 |
| Ecoli9000q_590 | High frequency lysogenization protein HflD | 3.055797576 |
| Ecoli9000q_47830 | Ferric uptake regulation protein | 3.043047177 |
| Ecoli9000q_13630 | Chain length determinant protein | 2.979668465 |
| Ecoli9000q_20830 | Pseudouridine synthase | 2.953027476 |
| Ecoli9000q_26570 | Toxin YhaV | 2.943455374 |
| Ecoli9000q_31870 | Lipopolysaccharide core biosynthesis protein rfaY | 2.925611269 |
| Ecoli9000q_4420 | N-acetyltransferase YncA | 2.857491414 |
| Ecoli9000q_33430 | hypothetical protein | 2.83383681 |
| Ecoli9000q_9660 | hypothetical protein | 2.813303769 |
| Ecoli9000q_26220 | Uronate isomerase | 2.802230473 |
| Ecoli9000q_35930 | HTH-type transcriptional repressor fabR | 2.78749035 |
| Ecoli9000q_33450 | Phosphate transport system permease protein pstC | 2.741594751 |
| Ecoli9000q_28110 | Ribosomal RNA small subunit methyltransferase B | 2.735740471 |
| Ecoli9000q_45330 | Exodeoxyribonuclease 7 small subunit | 2.692729739 |
| Ecoli9000q_36870 | Eae protein | 2.653334792 |
| Ecoli9000q_19260 | Ethanolamine utilization protein EutL | 2.645832848 |
| Ecoli9000q_3140 | Exoribonuclease 2 | 2.608548239 |
| Ecoli9000q_54120 | Glucans biosynthesis glucosyltransferase H | 2.551367208 |
| Ecoli9000q_33400 | hypothetical protein | 2.534602918 |
| Ecoli9000q_38270 | N-acetylmuramoyl-L-alanine amidase AmiB | 2.531975021 |
| Ecoli9000q_34250 | site-specific tyrosine recombinase XerC | 2.531000412 |
| Ecoli9000q_54490 | protein yceD | 2.516521899 |
| Ecoli9000q_1500 | Orf2 | 2.485792275 |
| Ecoli9000q_34530 | hypothetical protein | 2.471861812 |
| Ecoli9000q_52250 | lipoprotein gfcB | 2.456749205 |
| Ecoli9000q_20320 | protein yphB | 2.433031518 |
| Ecoli9000q_46180 | ABC transporter ATP-binding protein YbbL | 2.428810586 |
| Ecoli9000q_24940 | hypothetical protein | 2.381542204 |
| Ecoli9000q_54700 | NADH dehydrogenase | 2.373597462 |
| Ecoli9000q_33520 | LpfA | 2.371276382 |
| Ecoli9000q_50640 | Thioredoxin reductase | 2.361072232 |
| Ecoli9000q_19110 | Sulfate transport system permease protein CysT | 2.33178553 |
| Ecoli9000q_34550 | Sec-independent protein translocase protein TatC | 2.307195545 |
| Ecoli9000q_52220 | Polysaccharide export protein | 2.303139092 |
| Ecoli9000q_14020 | Protein AsmA | 2.300268445 |
| Ecoli9000q_29470 | ATP-dependent DNA helicase, RecQ | 2.286268896 |
| Ecoli9000q_38600 | hypothetical protein | 2.259280167 |
| Ecoli9000q_7610 | Protein ydgH | 2.225616921 |
| Ecoli9000q_10090 | protein yebW | 2.219130252 |
| Ecoli9000q_39720 | HTH-type transcriptional regulator QseD | 2.213357336 |
| Ecoli9000q_52470 | reductase | 2.208746184 |
| Ecoli9000q_35230 | Rhamnulose-1-phosphate aldolase | 2.203717425 |
| Ecoli9000q_27780 | RNase E specificity factor CsrD | 2.195074895 |
| Ecoli9000q_31910 | Lipopolysaccharide core heptosyltransferase rfaQ | 2.185876908 |
| Ecoli9000q_42590 | hypothetical protein | 2.143596558 |
| Ecoli9000q_49490 | ATP-dependent helicase dinG | 2.134458526 |
| Ecoli9000q_36740 | LexA repressor | 2.126132397 |
| Ecoli9000q_38280 | DNA mismatch repair protein mutL | 2.124632142 |
| Ecoli9000q_29630 | Tetratricopeptide TPR_2 repeat protein | 2.120902593 |
| Ecoli9000q_30030 | FabF | 2.106471489 |
| Ecoli9000q_52210 | Low molecular weight protein-tyrosine-phosphatase etp | 2.093210765 |
| Ecoli9000q_27380 | hypothetical protein | 2.08572709 |
| Ecoli9000q_34190 | Adenylate cyclase | 2.06611786 |
| Ecoli9000q_19690 | Hydrogenase-4 component I | 2.061788739 |
| Ecoli9000q_1950 | hypothetical protein | 2.060994087 |
| Ecoli9000q_860 | Portal protein B | 2.057171473 |
| Ecoli9000q_26270 | Protein yqjC | 2.035797293 |
| Ecoli9000q_14530 | molybdate metabolism regulator | 2.001034647 |
