# Supplementary material for: Transposon Insertion Sequencing Elucidates Novel Gene Involvement in Susceptibility and Resistance to Phages T4 and T7 in Escherichia coli O157
Source: mBio. 2018 Jul 24;9(4):e00705-18. doi: 10.1128/mBio.00705-18 (PMC6058288; doi:10.1128/mBio.00705-18)
Supplement: TABLE S2 [file mbo004183993st2.docx]

| Gene Locus | Annotation | LogFC vs the control |
| --- | --- | --- |
| Ecoli9000q_27560 | Stringent starvation protein A | -9.544824 |
| Ecoli9000q_48600 | UDP-galactose 4-epimerase | -8.2397784 |
| Ecoli9000q_13690 | GDP-L-fucose synthetase | -8.1276917 |
| Ecoli9000q_13710 | Glycosyl transferase | -7.6186096 |
| Ecoli9000q_13670 | Mannose-1-phosphate guanylyltransferase 2 | -6.9297282 |
| Ecoli9000q_13780 | UDP-N-acetylglucosamine 4-epimerase | -6.5629502 |
| Ecoli9000q_13700 | GDP-mannose 4,6-dehydratase | -5.9619472 |
| Ecoli9000q_31840 | O-antigen ligase | -5.9264937 |
| Ecoli9000q_13760 | Glycosyl transferase | -5.6220736 |
| Ecoli9000q_38970 | Trehalose-6-phosphate hydrolase | -5.5770472 |
| Ecoli9000q_24770 | hypothetical protein | -5.4304707 |
| Ecoli9000q_13680 | hydrolase | -5.2404962 |
| Ecoli9000q_36540 | Glucose-6-phosphate isomerase | -5.1642333 |
| Ecoli9000q_33990 | Undecaprenyl-phosphate alpha-N-acetylglucosaminyl 1-phosphate transferase | -4.9925453 |
| Ecoli9000q_13660 | Phosphomannomutase | -4.7959857 |
| Ecoli9000q_13750 | O antigen polymerase Wzy | -4.7737894 |
| Ecoli9000q_27870 | tRNA-dihydrouridine synthase B | -4.5789061 |
| Ecoli9000q_31850 | Lipopolysaccharide 1,2-N-acetylglucosaminetransferase | -4.4548486 |
| Ecoli9000q_8610 | Ferredoxin-like protein ydiT | -4.1433984 |
| Ecoli9000q_31860 | UDP-glucose:(Galactosyl) LPS alpha1,2-glucosyltransferase WaaJ | -3.7463749 |
| Ecoli9000q_30380 | Glutathione-disulfide reductase | -3.6298878 |
| Ecoli9000q_34070 | Protein wzxE | -3.5227328 |
| Ecoli9000q_13720 | Perosamine synthetase Per | -3.486645 |
| Ecoli9000q_27140 | Transcription elongation factor greA | -3.3627566 |
| Ecoli9000q_31590 | Mannitol-1-phosphate 5-dehydrogenase | -3.2612266 |
| Ecoli9000q_17390 | 50S ribosomal protein L3 glutamine methyltransferase | -3.1960353 |
| Ecoli9000q_13770 | UDP-glucose pyrophosphorylase | -3.0742415 |
| Ecoli9000q_6710 | Exodeoxyribonuclease VIII from bacteriophage origin | -2.9339729 |
| Ecoli9000q_48970 | LF82 chromosome, complete sequence | -2.8687045 |
| Ecoli9000q_38290 | tRNA dimethylallyltransferase | -2.8565052 |
| Ecoli9000q_15240 | Conserved predicted protein | -2.8528854 |
| Ecoli9000q_54750 | Transcription-repair-coupling factor | -2.820876 |
| Ecoli9000q_25700 | Outer membrane protein tolC | -2.7309239 |
| Ecoli9000q_10040 | Protein yebR | -2.6647867 |
| Ecoli9000q_45220 | lipoprotein yajI | -2.5525341 |
| Ecoli9000q_12210 | hypothetical protein | -2.4230068 |
| Ecoli9000q_40650 | Chaperone protein DnaJ | -2.4227901 |
| Ecoli9000q_53350 | L0014-like protein | -2.1913151 |
| Ecoli9000q_6540 | hypothetical protein | -2.1265513 |
| Ecoli9000q_49690 | L,D-transpeptidase YbiS | -2.095896 |
| Ecoli9000q_t190 | tRNA | -2.0932497 |
| Ecoli9000q_47740 | N-acetylglucosamine-6-phosphate deacetylase | -2.0779462 |
| Ecoli9000q_6510 | Antitermination protein | -2.0665247 |
| Ecoli9000q_22540 | hypothetical protein | -2.0457362 |
