# Supplementary figures and images for: Transposon Insertion Sequencing Elucidates Novel Gene Involvement in Susceptibility and Resistance to Phages T4 and T7 in Escherichia coli O157
Source: mBio. 2018 Jul 24;9(4):e00705-18. doi: 10.1128/mBio.00705-18 (PMC6058288; doi:10.1128/mBio.00705-18)

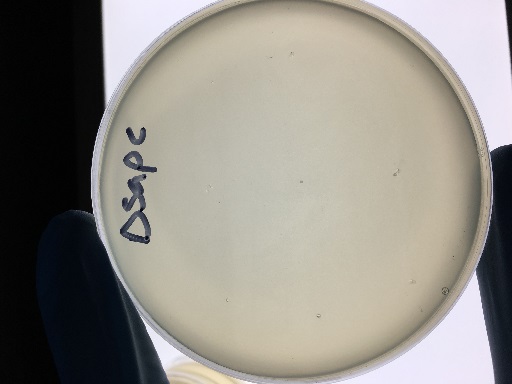


Δ*sapC*

**-**


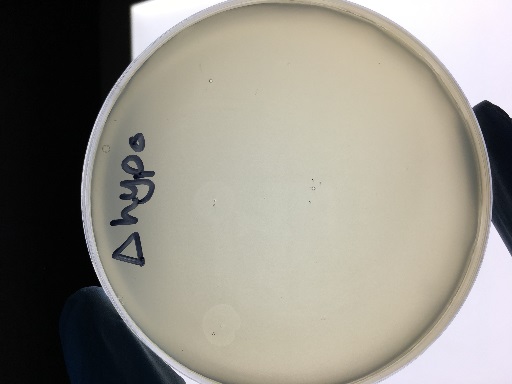


ΔEcoli9000q_26980

**(+)**


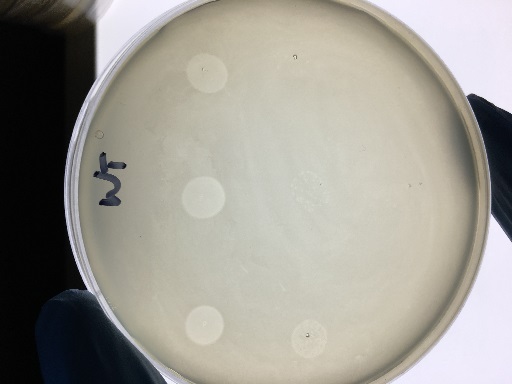


1465

**+++(+)**


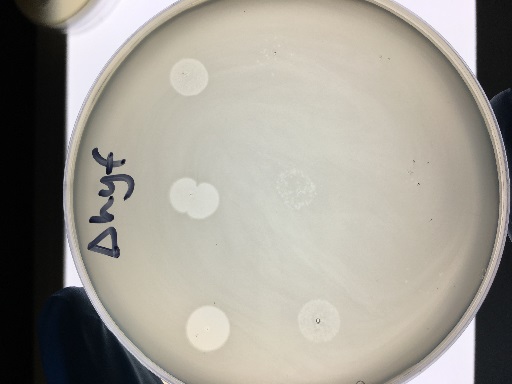


Δ*hyfR*

**+++(+)**


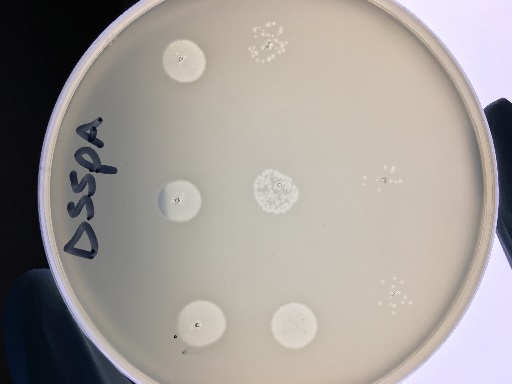


Δ*sspA*

**++++(+)(+)**


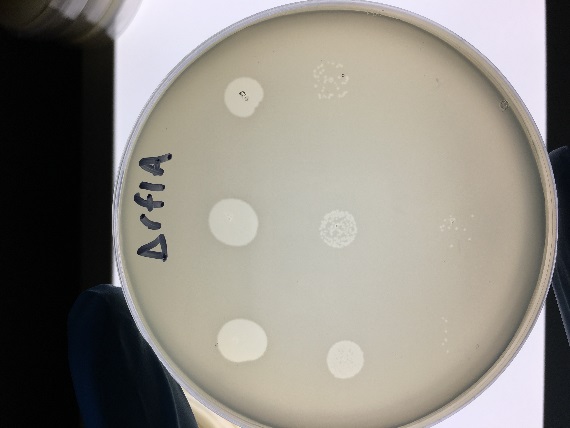


Δ*rflA*

**++++(+)(+)**


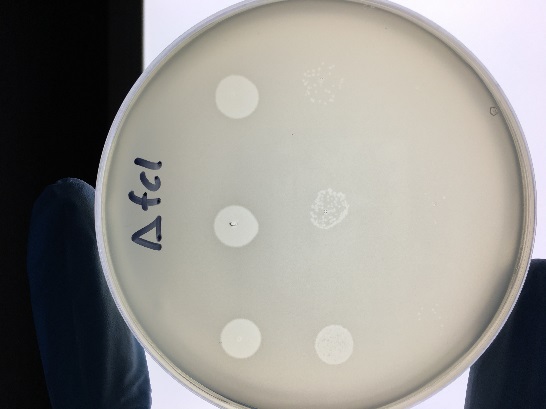


Δ*fcl*

**++++(+)**

Supplement: FIG S1 [file mbo004183993sf1.docx]
