# Supplementary material for: Transposon Insertion Sequencing Elucidates Novel Gene Involvement in Susceptibility and Resistance to Phages T4 and T7 in Escherichia coli O157
Source: mBio. 2018 Jul 24;9(4):e00705-18. doi: 10.1128/mBio.00705-18 (PMC6058288; doi:10.1128/mBio.00705-18)
Supplement: TABLE S3 [file mbo004183993st3.docx]

| Gene | Primer Name | Primer Sequence |
| --- | --- | --- |
| EzTn5 | **5' PCR primer sequence** | AATGATACGGCGACCACCGAGATCTACACATGATGATATATTTTTATCTTGTGCAATGTAACATCAGAG |
|  | **3' PCR primer sequence** | AATGATACGGCGACCACCGAGATCTACACAACAAAGCTCTCATCAACCGTGGC |
|  | **5' Sequencing primer sequence** | TGAGACACAATTCATCGATGATGGTTGAGATGTGTA |
|  | **3' Sequencing primer sequence** | CTGCAGGCATGCAAGCTTCAGGGTTGAGATGTGTA |
