# Supplementary material for: Transposon Insertion Sequencing Elucidates Novel Gene Involvement in Susceptibility and Resistance to Phages T4 and T7 in Escherichia coli O157
Source: mBio. 2018 Jul 24;9(4):e00705-18. doi: 10.1128/mBio.00705-18 (PMC6058288; doi:10.1128/mBio.00705-18)
Supplement: TABLE S4 [file mbo004183993st4.docx]

| Gene | Function | Primer Name | Primer Sequence |
| --- | --- | --- | --- |
| *sspA* | Stringent starvation protein A | sspAP1 | AACACGTGGAAAAGGACAATCCGCCTCAGGATCTGATTGACCTCAGTGTAGGCTGGAGCTGCTTC |
|  |  | sspAP2 | CAGACGCATTTCACGTTCTGCTTCAGTTAAAGAAGCAAGGAAAGACATATGAATATCCTCCTTAG |
|  |  | sspAtest | CGAACAAAGAGAGTCGTTCCT |
| *fcl* | GDP-L-fucose synthetase | GDPP1 | GAATAAGCAACGTATTTTTATTGCTGGTCACCAAGGAATGGTTGGGTGTAGGCTGGAGCTGCTTC |
|  |  | GDPP2 | TTCAAGACCCTTGTGAAGGGTAATTTTATGATTCCAACCTAGTTGCATATGAATATCCTCCTTAG |
|  |  | GDPtest | GCTGAAATGATTTCTGAAATGG |
| *fhlA* | Hydrogenase-4 transcriptional activator | hyfP1 | CAGAAAAATTGCGTGAGAAGGATTTCTCATTAATAAGGACTGTTGGTGTAGGCTGGAGCTGCTTC |
|  |  | hyfP2 | GTCGAAAGAGAGTTTGTTTCTCATGCATACTCCTGCAAAAGCAGACATATGAATATCCTCCTTAG |
|  |  | hyftest | GGATGAAATTGTGCTGGAACC |
| *-* | Hypothetical protein | hypoP1 | AGGCGTCCTGCCCGGTTAAAAGCCCCCCGCCGCAGCGGAGGGCAAGTGTAGGCTGGAGCTGCTTC |
|  |  | hypoP2 | ATGCGCAGAAGATCGGGTATTAACACCAGTGCCGTAAGGTACTGTCATATGAATATCCTCCTTAG |
|  |  | hypotest | CCACGGTTGAATGAACGTCC |
| *sapC* | sapC | sapCP1 | ATATTTTGGGTGCCATGGCTAACCCTCTGAAACATAAGGAATGGTGTGTAGGCTGGAGCTGCTTC |
|  |  | sapCP2 | CGGTTTTAAATTCAATAGTCAGGTTACGAATATCGAGTAATGGCACATATGAATATCCTCCTTAG |
|  |  | sapCtest | CCGGAGTGATGGTGTGTGG |
| *waaL* | O antigen ligase | rflAP1 | ATGACCTCAACATTATTTTTCTCTCTCGAGAAAAAAAACTGGATAGTGTAGGCTGGAGCTGCTTC |
|  |  | rflAP2 | TATATAATATGCATTATATGGTGAGCGATATTTATTCTTGACGCCCATATGAATATCCTCCTTAG |
|  |  | rflAtest | CGTTCTACAAGTATGTCTC |
| *Cm* | Chloramphenicol resistance | C1 | TTATACGCAAGGCGACAAGG |
